# Magnesium stearate, a widely-used food additive, exhibits a lack of *in vitro* and *in vivo* genotoxic potential

**DOI:** 10.1016/j.toxrep.2017.10.003

**Published:** 2017-10-16

**Authors:** Cheryl A. Hobbs, Kazuhiko Saigo, Mihoko Koyanagi, Shim-mo Hayashi

**Affiliations:** a Toxicology Program, Integrated Laboratory Systems, Inc., PO Box 13501, Research Triangle Park, NC 27709, USA; b Drug Safety Research Laboratories, Shin Nippon Biomedical Laboratories, Ltd., 2438 Miyanoura-cho, Kagoshima-City, Kagoshima 891-1394, Japan; c Global Scientific and Regulatory Affairs, San-Ei Gen F.F.I., Inc., 1-1-11 Sanwa-cho, Toyonaka, Osaka 561-8588, Japan

**Keywords:** 2AA, 2-aminoanthracene, 9AA, 9-aminoacridine hydrochloride monohydrate, ADI, acceptable daily intake, AF-2, 2-(2-furyl)-3-(5-nitro-2-furyl) acrylamide, DMSO, dimethyl sulfoxide, EFSA, European Food Safety Authority, FAO, Food and Agriculture Organization of the United Nations, ENNG, *N*-ethyl-*N*'-nitro-*N*-nitrosoguanidine, FDA, U.S. Food and Drug Administration, GLP, Good Laboratory Practice, JECFA, Joint FAO/WHO Expert Committee on Food Additives, MMC, mitomycin C, MN, micronucleus or micronuclei, MN-PCE, micronucleated polychromatic erythrocyte(s), OECD, Organization for Economic Cooperation and Development, PCE, polychromatic erythrocyte(s), WHO, World Health Organization, Genotoxicity, Food additive, Magnesium stearate, DNA damage, Dietary supplement, Joint FAO/WHO Expert Committee on Food Additives (JECFA)

## Abstract

Magnesium stearate is widely used in the production of dietary supplement and pharmaceutical tablets, capsules and powders as well as many food products, including a variety of confectionery, spices and baking ingredients. Although considered to have a safe toxicity profile, there is no available information regarding its potential to induce genetic toxicity. To aid safety assessment efforts, magnesium sulfate was evaluated in a battery of tests including a bacterial reverse mutation assay, an *in vitro* chromosome aberration assay, and an *in vivo* erythrocyte micronucleus assay. Magnesium stearate did not produce a positive response in any of the five bacterial strains tested, in the absence or presence of metabolic activation. Similarly, exposure to magnesium stearate did not lead to chromosomal aberrations in CHL/IU Chinese hamster lung fibroblasts, with or without metabolic activation, or induce micronuclei in the bone marrow of male CD-1 mice. These studies have been used by the Japanese government and the Joint FAO/WHO Expert Committee on Food Additives in their respective safety assessments of magnesium stearate. These data indicate a lack of genotoxic risk posed by magnesium stearate consumed at current estimated dietary exposures. However, health effects of cumulative exposure to magnesium via multiple sources present in food additives may be of concern and warrant further evaluation.

## Highlights

• Magnesium stearate is widely used as a food additive.
• No published data available related to magnesium stearate genotoxic potential.
• Magnesium stearate was evaluated in a battery of genetic toxicity tests.
• Ames, *in vitro* chromosome aberration and mouse micronucleus assays were negative.
• Results provided the basis for the genotoxicity assessment conducted by JECFA.

## Graphical abstract

**Figure:**
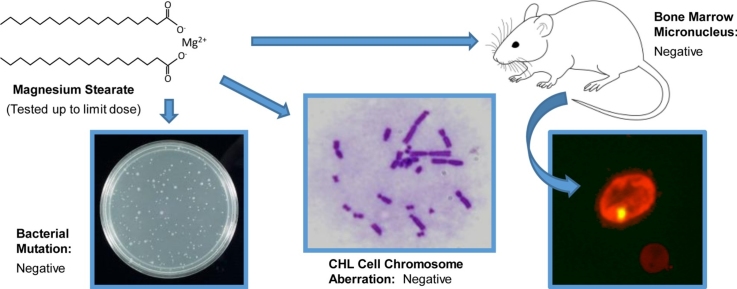

## 1 Introduction

**Fig. 1:**
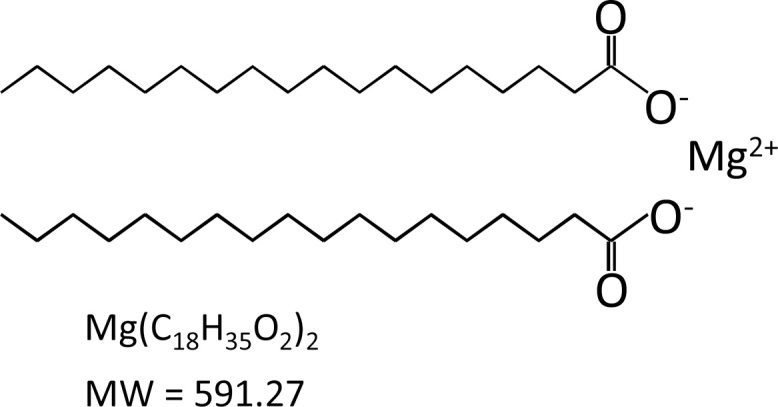
Chemical structure of magnesium stearate. Magnesium stearate, also known as octadecanoic acid, exists as a salt containing two stearate anions and a magnesium cation.

Magnesium stearate is the magnesium salt of the fatty acid, stearic acid (Fig. 1). It has been widely used for many decades in the food industry as an emulsifier, binder and thickener, as well as an anticaking, lubricant, release, and antifoaming agent. It is present in many food supplements, confectionery, chewing gum, herbs and spices, and baking ingredients. Magnesium stearate is also commonly used as an inactive ingredient in the production of pharmaceutical tablets, capsules and powders.

For food applications, magnesium stearate is typically manufactured by one of two processes. The direct or fusion process involves direct reaction of fatty acids with a source of magnesium, such as magnesium oxide, to form magnesium salts of the fatty acids. In the indirect or precipitation process, a sodium soap is produced by reacting fatty acids with sodium hydroxide in water and precipitating the product through addition of magnesium salts to the soap. The fatty acids used as raw material are derived from edible fats and oils and consist mainly of stearic and palmitic acid. The final product contains 4.0-5.0% magnesium, on a dried basis, and the fatty acid fraction is composed of ≥90% stearic and palmitic acids, at least 40% of which are stearic acid. It is a very fine powder that is greasy to the touch and practically insoluble in water.

Upon ingestion, magnesium stearate is dissolved into magnesium ion and stearic and palmitic acids. Magnesium is absorbed primarily in the small intestine, and to a lesser extent, in the colon. Magnesium is an essential mineral, serving as a cofactor for hundreds of enzymatic reactions and is essential for the synthesis of carbohydrates, lipids, nucleic acids and proteins, as well as neuromuscular and cardiovascular function [1], [2]. The majority of magnesium content in the body is stored in bone and muscle [1], [3]. A small amount (∼1%) is present in serum and interstitial body fluid, mostly existing as a free cation while the remainder is bound to protein or exists as anion complexes [3]. The kidney is largely responsible for magnesium homeostasis and maintenance of serum concentration [1], [3]. Excretion occurs primarily via the urine, but also occurs in sweat and breast milk. Stearic and palmitic acids are products of the metabolism of edible oils and fats for which the metabolic fate has been well established. These fatty acids undergo ß-oxidation to yield 2-carbon units which enter the tricarboxylic acid cycle and the metabolic products are utilized and excreted [4].

Magnesium stearate is permitted for use in the European Union and other countries including China, Japan, Australia and New Zealand, and was granted generally recognized as safe (GRAS) status in the United States [5]. However, there are no published data available related to the genotoxic potential of magnesium stearate. For the safety assessment of food ingredients, the U.S. Food and Drug Administration (FDA) recommends a bacterial reverse mutation test [6], [7], [8], an *in vitro* test for chromosomal damage or gene mutation in mammalian cells, as well as an *in vivo* test for chromosomal damage using mammalian hematopoietic cells [9], such as the rodent erythrocyte micronucleus assay [10], [11] which has proven utility for predicting carcinogens [12]. The European Food Safety Authority (EFSA) guidances [13], [14] recommend a similar, albeit tiered, approach for assessing genotoxic potential. To provide requested genotoxicity data to support initial assessment of the safety of magnesium stearate conducted by the Japanese government, and subsequently, reassessment by the Joint FAO/WHO Expert Committee on Food Additives [15], [16], magnesium stearate was evaluated in a bacterial gene mutation assay using *Salmonella* and *E. coli* tester strains, an *in vitro* mammalian chromosome aberration assay using Chinese Hamster Lung cells and a micronucleus assay in the bone marrow of male CD-1 mice. This combination of tests is commonly used to evaluate the genotoxicity of food additives [17], [18]. The studies reported here were performed as Good Laboratory Practice (GLP) experiments in accordance with Japanese Ministry of Health, Labour and Welfare and OECD testing guidelines current at the time the studies were conducted [19], [20], [21], [22].

## 2 Material and methods

### 2.1 Chemicals

All genotoxicity assays were GLP-compliant; however, analysis of dose formulations for concentration was not mandated by the Japanese regulatory agency requesting these studies and was not performed. Magnesium stearate (99% relative content of stearic and palmitic acid; CAS No. 557-04-0; San-Ei Gen F.F.I., Inc., Osaka, Japan) was stored at room temperature. Formulations were prepared just prior to use by adding vehicle to the weighed test substance and solubilizing with ultrasound; lower concentrations were prepared by serial dilution. Dimethyl sulfoxide (DMSO) was purchased from Sigma-Aldrich Japan K.K. (Shinagawa-ku, Japan). 2-(2-Furyl)-3-(5-nitro-2-furyl) acrylamide (AF-2), 2-aminoanthracene (2AA), sodium carboxymethyl cellulose, and mitomycin C (MMC) were purchased from Wako Pure Chemical Industries, Ltd., Osaka, Japan. 9-Aminoacridine hydrochloride monohydrate (9AA) and *N*-ethyl-*N'*-nitro-*N*-nitrosoguanidine (ENNG) were purchased from Nacalai Tesque, Inc. (Kyoto, Japan). Japanese Pharmacopeia saline was purchased from the Otsuka Pharmaceutical Factory, Inc. (Tokushima, Japan).

### 2.2 Bacterial reverse mutation assay

A bacterial mutagenicity assay of magnesium stearate, with and without metabolic activation, was conducted using the preincubation method using *Salmonella typhimurium* strains TA100 and TA1535 and *Escherichia coli* strain WP2*uvr* A as detection systems for base-pair substitution mutations, and *S. typhimurium* strains TA98 and TA1537 for detection of frame-shift mutations [6], [7], [8]. All strains (National Institute of Health Sciences, Japan) were checked for maintenance of genetic markers prior to the study. Based on results of a range-finding assay using all tester strains at 2 plates per concentration (Supplemental Data Table S1), a top concentration of 5 mg/plate, with and without metabolic activation, was chosen as recommended by expert group [7] as well as OECD [21] and Japanese [22] guidelines for non-cytotoxic compounds. The doses tested were 5000, 2500, 1250, 625, 313, and 156 μg/plate. Strain specific positive controls tested without metabolic activation were AF-2 (TA98 and TA100), ENNG (TA1535 and *E. coli* WP2), and 9AA (TA1537). 2-AA was used as the positive control for all strains tested with metabolic activation. Metabolic activation was provided by a 10% phenobarbital/5,6-benzoflavone-induced rat liver S9 preparation (Kikkoman Corp., Co., Noda, Japan) with added cofactors (glucose-6-phosphate dehydrogenase, NADPH, and NADH, Oriental Yeast Co., Ltd, Tokyo, Japan). Test solutions were prepared in DMSO. The assay tubes were pre-incubated at 37 °C for 20 min with shaking before addition of top agar and plating onto minimal agar. Two test plates per concentration were inverted and cultured at 37 °C for 48 h and then revertant colonies counted using an automatic colony counter (CA-7A, Toyo Sokki Co., Ltd., Japan). The use of two plates for all the tester strains with no evidence of a positive response in the rangefinder assay justifies the use of only two plates per concentration in the definitive assay, in accordance with the OECD test guideline that states that duplicate plating is acceptable when scientifically justified; this study design is also acceptable to the Japanese regulatory authority. Appropriate control plates were included to verify sterility of the vehicle, test chemical solutions, and S9 mix. Criteria for a positive response were a ≥2-fold increase in the average plate count compared to the vehicle control for at least one concentration level, a dose response over the range of tested concentrations in at least one strain with or without metabolic activation, and reproducibility between the range finding and definitive mutagenicity studies.

### 2.3 *In vitro* chromosome aberration assay

The mycoplasma-free CHL/IU Chinese hamster lung fibroblast cell line was obtained from the Division of Laboratory Products, Dainippon Pharmaceutical Co., Ltd. This cell line has an approximate cell doubling time of 15 h. Cells were cultured in Eagle MEM medium containing 10% heat inactivated fetal bovine serum at 37 °C with 5% CO_2_ and high humidity. S9 liver homogenate, prepared from male rats treated with phenobarbital and 5,6-benzoflavone (Kikkoman Corporation, Noda, Japan), was added at a final concentration of 30% to a filtered coenzyme solution containing 1.7 mg/mL glucose-6-phosphate dehydrogenase, 3.35 mg/mL NADPH, 4 mM HEPES, 5 mM MgCl_2_·6H_2_O, and 33 mM KCl. The assay was performed for short-term (6-h) and continuous treatments as described previously [23], [24] and in accordance with JMHLW guidelines current at the time. Based on the results of a range finding study of magnesium stearate (Supplemental Data Table S2), the 50% growth-inhibitory concentrations were estimated to be 49 μg/mL for the short-term treatment without metabolic activation, 784 μg/mL for the short-term treatment with metabolic activation, 9 μg/mL for the continuous 24-h treatment, and 4 μg/mL for the continuous 48-h treatment. The top concentrations of magnesium stearate selected for the chromosomal aberration test were 1000 and 50 μg/mL for the short-term treatment with and without metabolic activation, respectively, and 10 and 5 μg/mL for the continuous 24- and 48-h treatments, respectively; 0.5% sodium carboxymethyl cellulose was used as the vehicle.

Freshly thawed cells were cultured for 72 h, then diluted to 1 × 10^4^ cells/mL; 5 mL of the suspension were transferred to each of two 6-cm plastic Petri dishes per treatment group, and cultured for 72 h. Then, 2.5 mL of the culture medium were removed from each petri dish and 0.5 mL S9 mix (final concentration of 5%) or culture medium was added for tests with and without metabolic activation, respectively. The final volume of vehicle, magnesium stearate, or MMC (20 μg/mL final concentration) formulations added to culture medium was 10%; B[a]P was added at 0.5% (0.15 μg/mL final concentration). After culturing for 6 h, the cells were rinsed once with physiological saline, 5 mL of fresh medium added, and cells cultured for an additional 18 h. For continuous exposures, 72 h after the start of the culture, 0.5 mL of the magnesium stearate formulation, vehicle, or MMC solution (final concentration of 0.05 μg/mL) was added and the cells were cultured for 24 or 48 h. Colcemid was added to each petri dish at a final concentration of 0.1 μg/mL 2 h before the end of the culture period.

Following treatment, cells were trypsinized and viability determined using trypan blue exclusion. The cell suspension was centrifuged at 1000 rpm for 5 min, and hypotonic treatment was performed by adding 5 mL of 0.075 M KCl at 37 °C to the cell pellet and incubating at 37 °C for 30 min. Next, 0.5 mL of chilled Carnoy's fixative (3:1 ratio of methanol and glacial acetic acid) was added the cells and the mixture was centrifuged (1000 rpm for 5 min). Approximately 4 mL of fixative was added to the cell pellet and the mixture was centrifuged; this cycle was repeated 3 times. Two drops of the fixed cell suspension were applied to each clean slide. These slides were air dried, stained with 3.0% Giemsa pH 6.8 (Merck, Kenilworth, NJ) for 15 min, rinsed in water, and allowed to dry. The slides were coded in a double-blind manner and one hundred metaphase spreads were observed per slide at magnifications up to 600×. Structural and numerical aberrations observed in cells with 25 ± 2 chromosomes were recorded. Structural aberrations were classified as a chromatid break, chromatid exchange, chromosome break, chromosome exchange, and others in accordance with the “Atlas on Chromosomal Aberrations” [25]. Interchromosomal exchanges (triradial or quadriradial) and intrachromosomal exchanges (ring chromatid) were recorded as chromatid exchanges, and dicentric chromosomes and ring chromosomes were recorded as chromosome exchanges. Many gaps (chromatid or chromosome) and breaks were considered as fragmentation, and 10 or more breaks and exchanges were considered as multiple aberrations, both of which were classified as “other” aberrations. If two or more aberrations were observed in a single cell, each of the aberrations was counted as a single aberration. Gaps were not included in the tabulation of structural aberrations. A gap was defined as an achromatic region that was narrower than the chromatid width, and a break was defined as an achromatic region that was wider than the chromatid width. For evaluation of numerical aberrations, metaphase spreads with ≥38 chromosomes were considered as polyploids and distinguished from endoreduplication. Eagle MEM, fetal bovine serum and colcemid were purchased from Gibco BRL (Grand Island, NY).

### 2.4 Animal husbandry

Male Crj: CD-1 (ICR) mice (Charles River Laboratories Japan, Inc.) were 7 weeks of age at the time of treatment. Animals were housed in aluminum cages with absorbent bedding (White Flakes, Charles River Laboratories Japan, Yokohama, Japan) in a specific pathogen free facility with a 12-h light/12-h dark cycle. Mice were provided cobalt-60 irradiated solid feed (CE-2, CLEA Japan, Inc., Tokyo, Japan) and water *ad libitum*.

### 2.5 *In vivo* erythrocyte micronucleus (MN) assay

In a dose range finding study (Supplemental Data Table S3), no death was observed at doses up to 2000 mg/kg magnesium stearate. The frequency of micronucleated polychromatic erythrocytes (MN-PCE) was normal in the bone marrow at 24, 48, and 72 h in all groups. The percentage of polychromatic erythrocytes (PCE) decreased in a dose-dependent manner 24 h, and to a lesser extent, 48 h, following administration of magnesium stearate without excessive cytotoxicity. Therefore, for the definitive study, male CD-1 mice (6 animals/dose group) were administered magnesium stearate at 2000, 1000, or 500 mg/kg or vehicle (0.5% sodium carboxymethyl cellulose) once by gastric tube, or the positive control compound, MMC in Japanese Pharmacopeia saline at 2 mg/kg, once by intraperitoneal injection. Twenty-four hours after administration, mice were sacrificed by cervical dislocation. Both femurs were removed, ends cut, and bone marrow cells flushed out with fetal bovine serum (FBS; GIBCO BRL, Grand Island, NY). The cells were centrifuged at 1000 rpm for 5 min and most of the supernatant was discarded. A few drops of the concentrated cell suspension were applied to a degreased slide, smeared using a cover glass, and allowed to dry at room temperature. The bone marrow slides were fixed in methanol for 5 min, stained for 30 min with a Giemsa stain that had been diluted to 3% with 6.7 mM sodium-potassium phosphate buffer (pH 6.8), and gently rinsed with sodium-potassium phosphate buffer. The slides were then treated with 0.004% citric acid solution for approximately 3 s, rinsed with distilled water, and allowed to dry.

The frequency of MN-PCE was determined by counting the number of micronuclei (MN) in 2000 PCE per animal using coded specimens and an oil immersion lens (final magnification: 1000×). Five hundred erythrocytes [PCE + normochromatic erythrocytes (NCE)] from each animal were scored to determine the percentage of PCE in total erythrocytes as an index of chemical-induced growth suppression of bone marrow cells.

### 2.6 Statistical analyses

A chi-square test (one-sided, *p *< 0.05) was used to compare the frequency of cells with chromosomal aberrations in each of the test substance groups with that in the vehicle control group; the test was considered positive if the frequency of cells with chromosomal aberrations was significantly increased and a dose dependency or reproducibility was observed. For the *in vivo* MN assay, significant differences in the frequency of MN-PCE between the vehicle control group, each of the magnesium stearate groups, and the positive control group were analyzed using the Kastenbaum and Bowman method [26]. If the frequency of MN-PCE in the test substance group was significantly higher than in the vehicle control group at a significance level of 5%, the test substance was considered to induce MN in mouse bone marrow cells. A Student's *t*-test was used to determine if the % PCE, an index of growth inhibition of bone marrow cells, was significantly different between a magnesium stearate-exposed group and the vehicle control group.

## 3 Results

### 3.1 Bacterial reverse mutation assay

A mutagenicity assay was conducted to assess the potential of magnesium stearate to induce gene mutations in bacteria up to the recommended maximum concentration for non-cytotoxic chemicals (5000 μg/plate). Growth inhibition of the test strains was not observed at any concentration; precipitation of magnesium stearate was observed at concentrations ≥313 μg/plate. Average plate counts for each set of replicate plates are provided in Table 1. Consistent with the results of the range finding assay (Supplemental Data Table S1), a positive mutagenic response to magnesium stearate was not produced in any of the five *Salmonella* or *E. coli* strains tested either with or without metabolic activation. Average revertant values for positive control chemicals, both with and without metabolic activation, were at least 2-fold above concurrent solvent controls. The numbers of revertant colonies in the vehicle and positive control groups were within the range of laboratory historical data. The lack of induction of an increase in revertant colonies or any apparent concentration-dependent response indicates that, under the assay conditions tested, magnesium stearate is not mutagenic in the bacterial reverse mutation assay.

**Table 1:** Results of bacterial reverse mutation assay of magnesium stearate.

Dose (μg/plate)	Mean revertants/plate (± SD) without rat liver S9					Mean revertants/plate (± SD) with rat liver S9				
	TA100	TA98	TA1535	TA1537	WP2 *uvrA*	TA100	TA98	TA1535	TA1537	WP2 *uvrA*
0	127 ± 4	27 ± 1	12 ± 0	8 ± 1	23 ± 3	138 ± 1	36 ± 3	13 ± 1	10 ± 1	22 ± 1
156	137 ± 1	34 ± 4	15 ± 4	5 ± 0	20 ± 1	148 ± 2	46 ± 1	17 ± 5	8 ± 5	18 ± 4
313	132 ± 10	29 ± 0	12 ± 1	5 ± 3	20 ± 4	147 ± 3	39 ± 5	12 ± 0	8 ± 4	21 ± 4
625	129 ± 8	33 ± 6	13 ± 0	6 ± 1	22 ± 4	132 ± 5	34 ± 6	16 ± 7	11 ± 1	19 ± 6
1250	132 ± 4	27 ± 8	11 ± 4	9 ± 1	18 ± 1	144 ± 1	47 ± 6	12 ± 2	10 ± 3	16 ± 1
2500	138 ± 11	33 ± 2	13 ± 3	11 ± 1	18 ± 5	143 ± 1	40 ± 2	14 ± 1	9 ± 2	22 ± 4
5000	130 ± 1	24 ± 1	15 ± 6	6 ± 1	21 ± 1	139 ± 0	44 ± 6	16 ± 5	10 ± 0	22 ± 3
										
Positive control	382 ± 13^a^	337 ± 6^b^	260 ± 14^c^	692 ± 62^d^	976 ± 49^e^	1238 ± 21^f^	647 ± 39^g^	241 ± 42^h^	222 ± 1^h^	232 ± 46^i^

^a^ 2-(2-Furyl)-3-(5-nitro-2-furyl)acrylamide administered at 0.01 μg/plate.

^b^ 2-(2-Furyl)-3-(5-nitro-2-furyl)acrylamide administered at 0.1 μg/plate.

^c^ N-Ethyl-N'-nitro-N-nitrosoguanidine administered at 5 μg/plate.

^d^ 9-Aminoacridine hydrochloride administered at 80 μg/plate.

^e^ N-Ethyl-N'-nitro-N-nitrosoguanidine administered at 2 μg/plate.

^f^ 2-Aminoanthracene administered at 1 μg/plate.

^g^ 2-Aminoanthracene administered at 0.5 μg/plate.

^h^ 2-Aminoanthracene administered at 2 μg/plate.

^i^ 2-Aminoanthracene administered at 10 μg/plate.

### 3.2 *In vitro* chromosome aberration assay

Precipitation was observed at the start and end of the treatment for magnesium stearate concentrations of 6.25 μg/mL and higher for short exposures and at doses of 5 μg/mL for continuous exposures. Excessive cytotoxicity precluded evaluation of chromosomal aberrations in cells exposed to 10 μg/mL for 24 h and 5 μg/mL for 48 h. Exposure to magnesium stearate did not induce increased frequencies of structural or numerical aberrations under any of the test conditions (Table 2). The positive control chemicals, MMC and B[a]P, induced statistically positive increases in the frequency of cells with structural, but not numerical, aberrations. Numerical aberrations would not be expected in response to MMC, a clastogen. B[a]P was observed to produce aneuploidy, but not polyploidy, at 2.5–10 μg/mL in V79-MZ Chinese hamster lung cells [27]; since only cells with 25 ± 2 chromosomes were scored in this study, aneuploidy would not have been detected even if induced at the much lower B[a]P concentration used in this study.

**Table 2:** Results of chromosome aberration assay in CHL cells exposed to magnesium stearate.

Dose (μg/mL)	Viability (%)	Structural Chromosomal Aberrations							Numerical Chromosome Aberrations	Endoreduplication	Total
	Mean	Chromatid Break	Chromatid Exchange	Chromosome Break	Chromosome Exchange	Others	Gaps^a^	Total^b^	Polyploid		
6 h Exposure without S9											
0	100.0	1	0	0	0	0	1	1	1	0	1
1.56	101.3	0	1	1	0	0	0	2	1	0	1
3.12	90.7	0	0	0	0	0	1	0	1	0	1
6.25^c^	78.8	2	0	1	0	0	0	3	2	0	2
12.5^c^	69.5	1	0	0	0	0	2	1	3	0	3
25^c^	53.6	0	1	1	0	0	1	2	3	0	3
50^c^	44.4	2	1	0	0	0	1	3	3	0	3
MMC (0.15)	62.3	11	40	0	0	0	0	43	0	0	0
6 h Exposure with S9											
0	100.0	1	1	0	0	0	0	2	2	0	2
31.3^c^	96.5	0	0	0	0	0	0	0	2	0	2
62.5^c^	90.0	0	1	1	0	0	1	2	3	0	3
125^c^	77.1	1	1	0	0	0	1	2	4	0	4
250^c^	62.9	0	0	0	0	0	1	0	4	0	4
500^c^	51.2	2	0	0	0	0	0	2	4	0	4
1000^c^	44.7	0	1	1	0	0	2	2	5	0	5
B(a)P (20)	45.9	7	46	0	0	0	0	50	0	0	0
24 h Exposure without S9											
0	100.0	0	1	0	0	0	1	1	1	0	1
0.313	94.0	1	0	1	0	0	0	2	1	0	1
0.625	83.3	2	0	0	0	0	1	2	2	0	2
1.25	64.3	0	1	0	0	0	1	1	3	0	3
2.5	53.6	1	0	1	0	0	1	2	2	0	2
5^c^	39.9	1	1	0	0	0	1	2	3	0	3
10	30.4	NA	NA	NA	NA	NA	NA	NA	NA	NA	NA
MMC (0.05)	69.0	15	44	0	0	0	0	49	0	0	0
48 h Exposure without S9											
0	100.0	1	0	0	0	0	1	1	2	0	2
0.156	94.9	1	1	0	0	0	0	2	2	0	2
0.313	87.0	1	0	0	0	0	1	1	3	0	3
0.625	71.8	1	0	1	0	0	1	2	3	0	3
1.25	57.9	0	1	0	0	0	2	1	3	0	3
2.5	45.4	0	1	0	0	0	1	1	3	0	3
5^c^	29.6	NA	NA	NA	NA	NA	NA	NA	NA	NA	NA
MMC (0.05)	56.0	11	49	0	0	0	0	54	0	0	0

NA = Not analyzed due to excessive cytotoxicity.

MMC = Mitomycin C; B(a)P = Benzo[a]pyrene.

^a^Gaps include both chromatid-type aberration and chromosome-type aberration.

^b^Gaps not included in total of structural aberrations.

^c^Precipitate observed.

### 3.3 *In vivo* MN assay

In a preliminary dose setting study, indication of chemical exposure without evidence of excessive cytotoxicity or MN induction was observed in the bone marrow of mice 24, 48, or 72 h following a single administration of magnesium stearate up to 2000 mg/kg (Supplemental Data Table S3). Based on these results, a MN assay was conducted in which male CD-1 mice were administered magnesium stearate orally once at 500, 1000, and 2000 mg/kg and bone marrow evaluated 24 h following chemical administration. The selection of a 24-h timepoint was in accordance with a published recommendation that if the frequency of MN-PCE did not increase significantly at any dose levels or sampling times tested up to 72 h in a dose setting study, the sampling time for the definitive study should be set at 24 or 30 h [28]. This study design was acceptable to the Japanese regulatory authority at the time the study was conducted [29]. All animals survived to termination. Results of analysis of MN-PCE and PCE frequencies are summarized in Table 3. Under the conditions used in the MN study, no increase in the frequency of MN-PCE was observed in mice administered magnesium stearate. Decreases in the % PCE relative to the vehicle control animals were measured in mice in all magnesium stearate dose groups, indicating some bone marrow cytotoxicity reflective of chemical exposure at the tested doses. There was a statistically significant increase in MN-PCE and suppression of PCE in the bone marrow of animals administered the concurrent positive control, MMC. The %MN-PCE and %PCE values for the vehicle and positive control groups fell within the laboratory historical control range.

**Table 3:** Results of micronucleus assay in mice administered magnesium stearate.

Dose (mg/kg)	% PCE^a^	%MN-PCE^a^
0	51.6 ± 2.1	0.10 ± 0.05
500	49.7 ± 2.2	0.11 ± 0.04
1000	48.7 ± 1.9^b^	0.09 ± 0.04
2000	43.6 ± 2.0^b^	0.11 ± 0.04
MMC	37.9 ± 2.7^b^	3.32 ± 0.30^c^

MMC = mitomycin C administered at 2 mg/kg.

^a^ Group mean ± standard deviation.

^b^ Significant at *p <* 0.05 (Student's *t*-test).

^c^ Significant at *p <* 0.05 (Kastenbaum and Bowman's method).

## 4 Discussion

These studies were conducted to produce genotoxicity information to aid safety assessment of magnesium stearate used as a food additive. In a bacterial reverse mutation assay, magnesium stearate did not produce a positive response in any of the five test strains, either with or without metabolic activation, up to the OECD-recommended limit dose. These results are consistent with results provided in a study report submitted to the FDA [30]. Likewise, exposure to magnesium stearate did not induce chromosomal aberrations in hamster lung fibroblasts or micronuclei in the bone marrow of CD-1 mice.

Upon ingestion, magnesium stearate dissolves into its component ions, magnesium and stearic and palmitic acids. Therefore, the safety assessment should be based on its constituent cations and anions. Fatty acids are normal constituents of coconut oil, butter and other edible oils and have not been considered to pose a toxicological risk [4], [31]. As such, it was concluded that stearic and palmitic acids used as flavouring agents do not present a safety concern [32]. This position has been supported by results of recent studies demonstrating a lack of genotoxicity and toxicity of some fatty acids containing stearic and palmitic acids [33], [34].

The results of the genetic toxicity tests reported here were used by the Japanese government in its assessment of the safety of magnesium stearate, leading to its approved use as a food additive for certain applications in Japan in 2006. The safety of magnesium stearate was most recently reviewed internationally at the 80th meeting of the Joint FAO/WHO Expert Committee on Food Additives in 2015 [15], [16]. The results of the studies reported here were provided to the Committee and served as the primary basis for its opinion that magnesium stearate is not genotoxic. The Committee also evaluated a range of other toxicological studies and assessed dietary exposure. It concluded that the toxicity of magnesium stearate should not be evaluated differently than other magnesium salts and confirmed the previously recommended [31] acceptable daily intake (ADI) of “not specified” for magnesium salts of stearic and palmitic acids. Subsequently, the Japanese Ministry of Health, Labour and Welfare conducted a re-evaluation of magnesium stearate, including the data from this genetic toxicity test battery, and expanded the existing use standards beyond foods for specified health uses and with nutrient function claims, to include foods not in conventional food form such as tablet confectioneries and capsules or tablets with functional claims (http://members.wto.org/crnattachments/2017/SPS/JPN/17_2276_00_e.pdf).

Toxicology data from animal studies relevant to evaluation of magnesium stearate are lacking (e.g., doses that won’t lead to a dietary imbalance, known composition of material tested, appropriate administration route, etc.) [15]. There are also no human data related to magnesium stearate toxicity. It has been noted that infants are particularly sensitive to the sedative effects of magnesium salts and that individuals with chronic renal impairment retained 15–30% of administered magnesium, which may cause toxicity [31]. Moreover, diarrhea and other gastrointestinal effects have been observed with excessive magnesium intake resulting from use of various magnesium salts for pharmacological/medicinal purposes. Many magnesium-containing food additives have been evaluated individually, but not collectively, for laxative effects. Based on the recent dietary exposure assessment to magnesium stearate and concern that use of magnesium salts in many food additives may result in cumulative exposure that could lead to a laxative effect, JECFA reiterated its earlier recommendation [35] that total dietary exposure to magnesium from food additives and other sources in the diet be assessed [15]. Although effects of cumulative exposure to magnesium via food additives should be evaluated, the studies reported here indicate a lack of genotoxic risk posed specifically by magnesium stearate consumed at current estimated dietary exposures.

## 5 Funding

This research did not receive any specific grant from funding agencies in the public, commercial, or not-for-profit sectors.
