# Supplementary material for: Magnesium stearate, a widely-used food additive, exhibits a lack of in vitro and in vivo genotoxic potential
Source: Toxicol Rep. 2017 Oct 16;4:554–9. doi: 10.1016/j.toxrep.2017.10.003 (PMC5655391; doi:10.1016/j.toxrep.2017.10.003)
Supplement: Supplementary file 1 [file mmc1.docx]

Supplemental Data Table S1. Bacterial reverse mutation assay results in range finding s

Concentration (μg/plate)	Without Metabolic Activation		With Metabolic Ac
	Revertants per Plate	Mean	Revertants per Plate
TA100			
Solvent	126, 126	126	116, 122
5	132, 137	135	141, 145
15	147, 144	146	131, 134
50	124, 140	132	128, 116
150	129, 127	128	127, 123
500	119, 121	120	125, 129
1500	119, 118	119	138, 127
5000	135, 110	123	141, 142
AF-2 (0.01)	348, 368	358	-
2AA (1)	-	-	1436, 1556
TA1535			
Solvent	15, 15	15	12, 16
5	15, 19	17	14, 12
15	11, 15	13	11, 9
50	9, 14	12	18, 12
150	12, 13	13	11, 10
500	17, 15	16	12, 17
1500	12, 11	12	16, 11
5000	13, 10	12	14, 13
ENNG (5)	276, 281	279	-
2AA (2)	-	-	326, 275
WP2*uvr* A			
Solvent	16, 18	17	18, 15
5	24, 16	20	20, 18
15	20, 22	21	17, 18
50	23, 21	22	27, 20
150	18, 17	18	23, 26
500	20, 22	21	24, 22
1500	15, 23	19	26, 22
5000	17, 21	19	18, 24
ENNG (2)	813, 796	805	-
2AA (10)	-	-	420, 362
TA98			
Solvent	20, 28	24	35, 35
5	30, 39	35	47, 41
15	37, 34	36	31, 47
50	22, 33	28	31, 33
150	35, 32	34	39, 39
500	39, 41	40	34, 40
1500	26, 31	29	46, 39
5000	25, 33	29	46, 41
AF-2 (0.1)	527, 486	507	-
2AA (0.5)	-	-	630, 595
TA1537			
Solvent	12, 7	10	10, 8
5	11, 7	9	13, 9
15	10, 7	9	13, 13
50	13, 10	12	8, 10
150	15, 13	14	14, 6
500	13, 7	10	5, 12
1500	9, 9	9	6, 9
5000	10, 9	10	9, 8
