# Supplementary material for: Magnesium stearate, a widely-used food additive, exhibits a lack of in vitro and in vivo genotoxic potential
Source: Toxicol Rep. 2017 Oct 16;4:554–9. doi: 10.1016/j.toxrep.2017.10.003 (PMC5655391; doi:10.1016/j.toxrep.2017.10.003)
Supplement: Supplementary file 2 [file mmc2.docx]

Supplemental Data Table S2: Results of range-finding study chromosome aberration assay of magnesium stearate

Concentration (µg/mL)	Viability (% of Control) # Mean
6-Hour exposure in the absence of metabolic activation	
0	100.0
5*	92.5
15*	82.5
50*	44.5
150*	23.0
500*	21.0
1500*	23.0
5000*	23.0
6-Hour exposure in the presence of metabolic activation	
0	100.0
5*	91.0
15*	89.0
50*	95.0
150*	85.0
500*	51.5
1500*	41.0
5000*	26.0
24-Hour exposure in the absence of metabolic activation	
0	100.0
5*	66.0
15*	32.0
50*	12.0
150*	16.0
500*	15.0
1500*	0.0
5000*	0.0
48-Hour exposure in the absence of metabolic activation	
0	100.0
5*	52.0
15*	10.0
50*	5.0
150*	0.0
500*	0.0
1500*	0.0
