# Supplementary material for: Magnesium stearate, a widely-used food additive, exhibits a lack of in vitro and in vivo genotoxic potential
Source: Toxicol Rep. 2017 Oct 16;4:554–9. doi: 10.1016/j.toxrep.2017.10.003 (PMC5655391; doi:10.1016/j.toxrep.2017.10.003)
Supplement: Supplementary file 3 [file mmc3.docx]

Supplemental Data Table S3. Results of preliminary micronucleus test of magnesium stearate

Concentration (mg/kg)	Endpoint	Sampling Time		
		24 Hours	48 Hours	72 Hours
31.3	%MN-PCE	0.05, 0.00	0.05, 0.10	0.05, 0.05
	%PCE	50.6, 48.0	47.6, 52.0	51.8, 54.0
125	%MN-PCE	0.10, 0.10	0.05, 0.05	0.10, 0.00
	%PCE	45.2, 46.0	54.4, 52.6	51.8, 49.6
500	%MN-PCE	0.0, 0.10	0.05, 0.05	0.10, 0.10
	%PCE	41.4, 49.0	49.2, 47.8	52.6, 50.2
2000	%MN-PCE	0.05, 0.10	0.05, 0.05	0.10, 0.10
	%PCE	41.8, 39.0	43.4, 45.2	49.8, 51.6

PCE = polychromatic erythrocytes; MN-PCE = micronucleated polychromatic erythrocytes
